# The squeaky wheel gets the grease: Violent civil unrest and global social assistance provision

**DOI:** 10.3389/fsoc.2022.891267

**Published:** 2022-10-05

**Authors:** Rahmi Çemen, Erdem Yörük

**Affiliations:** ^1^University of Florida, Gainesville, FL, United States; ^2^University of Oxford, Oxford, United Kingdom; ^3^Koç University, Istanbul, Turkey

**Keywords:** social assistance, social unrest, protest, welfare (social) state, Global South and globalization

## Abstract

What are the contemporary determinates of social assistance provision? What is the role of contentious politics? Social assistance literature is dominated by economic and demographic accounts, which under-examine the possibility that governments extend social assistance to contain social unrest. We test factors associated with these “structuralist” and “political” theories on a new panel dataset which includes 54 OECD and emerging market countries between 2002 and 2015. The results indicate social assistance coverage has a significant positive relationship with riots. We explain this outcome as policymakers expanding social assistance as a means of containing violent civil unrest. This effect is more significant in emerging markets, suggesting that the domination of structural explanations is a result of sample bias toward the OECD. Finally, we find that governments consider World Bank social policy recommendations only insofar as there is violent unrest.

## Introduction

Over the last two decades, welfare systems around the globe have experienced important changes in their scope and content. Especially in the Global South, one of the most significant recent developments has been the creation and reform of innovative new social assistance programs. This trend is led by the so-called emerging market economies (EME), which have introduced a vast number of inclusive social assistance programs providing cash and in-kind support for the poor (including conditional cash transfers, free healthcare, food aid, and public works programs). This has led the ILO (2014) to argue that a significant divergence is taking place between an eroding “European social model” and rising “inclusive growth through social protection” in emerging markets. In a similar vein, Minnite and Piven (2013) argue that a historical bifurcation is taking place in global welfare systems. While advanced capitalist countries have undergone significant welfare retrenchments that reduce the capacity to decommodify labor and level out inequalities, they argue that countries in the Global South have expanded their welfare states on the basis of novel expansive social assistance programs. In this article, we look for the key factors contributing to this trend by focusing on the effects of contentious politics.

We start with a review of the literature on the origins and transformations of welfare states. This review points to the bias in the contemporary welfare state literature toward what we call “structuralist” approaches (Korpi, 1980; Esping-Andersen, 1990). These approaches explain the origins and reform of welfare states as a “natural” response to a variety of demographic and economic exigencies, such as aging, unemployment, previous development strategies, and political institutions (Pierson, 2001; Rudra, 2002; Haggard and Kaufman, 2020). However, most popular contemporary theories of welfare state creation and transformation have difficulty explaining the recent dominant role of social assistance in emerging markets. While a variety of market and social forces have been theorized to explain the expansion of social insurance-based welfare (Korpi and Palme, 1998; Estevez-Abe, 1999), structuralist approaches leave little room for the kind of proliferation in social assistance taking place in 21st century EMEs.

Without denying the important contributions of structuralist approaches, we contend that the contemporary literature has largely under-examined the effect that political responses to social unrest have on social assistance provision. This line of inquiry is inspired by mid-20th century scholars who delicately fused structural and political perspectives to explain the creation and transformation of modern welfare states. According to these authors, socio-structural pressures are translated into social policies through political conflict and struggles. These scholars considered the mid-twentieth century welfare expansion as part of a strategy to contain political disorder and mobilize popular support (Dawson and Robinson, 1963; Jennings, 1979; Olson, 1982). For instance, in-depth investigations of the urban riots in the United States during the 1960s provide evidence of the state using welfare programs for controlling, containing, and potentially repressing insurgent populations (Cloward and Piven, 1971; Gurr, 1980; Isaac and Kelly, 1981; Offe, 2018). To date, few studies have inquired into whether similar processes might be shaping the contemporary welfare institutions of developed and developing countries.

In this study we set out to test whether social unrest is an important source of political pressure on governments to expand social assistance. In order to do so, we introduce a new cross-national panel dataset which includes 54 original OECD and emerging market economies between the years 2002–2015. The results of the panel data analysis show a significant positive relationship between social assistance coverage and riots. However, we find that other forms of peaceful unrest, such as general strikes and anti-government demonstrations, do not have a statistically significant effect on coverage. The relationship between riots and social assistance coverage remains robust after controlling for a variety of alternative explanations. We, therefore, propose that that political containment of violent disruption is an important motivating factor for the expansion of social assistance coverage, especially in emerging markets.

Our results are in line with the expectations of the mid-20th century welfare state scholars, who suggest policymakers are motivated by concerns with domestic political stability when formulating and reforming social welfare programs. Thus, we argue that the positive relationship between riots and social assistance is stims from the need of policymakers to expand social assistance coverage to (potentially) radicalized/insurgent poor population. This is expected to help bring legitimacy for governments by decreasing popular grievances and reducing the chances of political insurgency by subverting part of the motives. A recent literature, which is mostly based on in-depth case studies, supports these claims by providing evidence that concerns with political stability and social unrest are indeed an important motivating factor in the social policy decision-making process for several different EME governments (Yörük, 2012; Li and Walker, 2016; Fallov and Blad, 2018; Yörük et al., 2019). We take these results one step further by showing a consistent *ceteris paribus* positive relationship between riots and social assistance coverage on a large sample of countries, thus indicating the existence of an international correlation between social assistance and contentious politics.

The relationship between riots and social assistance coverage remains robust to a variety of different model specifications, including controlling for a number of alternative structural and international factors which might be influencing the results. For example, when riots precede a newly created World Bank social policy recommendations variable, the interaction term between social unrest and WBSPR is positive. We take this to indicate that World Bank social policy recommendations are taken into action by governments to the extent that these governments are challenged by violent social unrest. Lastly, we conduct a comparison of OECD and EME countries, which highlights the greater significance of riots for the sample of EMEs. We, therefore, cautiously raise the concern that the tendency of the contemporary welfare literature to focus on structural factors, with much less attention given to the political processes that translate structural pressures into actual social policy, may be partly due to a sample bias toward advanced industrialized democracies.

The remainder of the article continues as follows. We first present a brief account of the global expansion of social assistance over the past two decades. Then, we survey the existing literature on the causes of ongoing social assistance expansion, by illustrating that, unlike the earlier literatures on mid-20th century welfare expansion, the contemporary literature suffers from excessive structuralism and under-examines contentious political factors. The literature review informs the theoretical foundations of our hypotheses on the relationship between violent civil unrest and social assistance provision. We then describe our data and methods, followed by the results section, and finally the conclusions.

## Global social assistance transformations

In the post-war period up to the late 1970s, welfare systems in many countries in the Global South were structured around employment-based social security programs in limited fragmented corporatist systems that excluded the urban and rural informal poor (Haggard and Kaufman, 2008). Although many social assistance programs have origins dating back much farther than the 1970s and 1980s, since that point many employment-based social security programs have been gradually retrenched while social assistance programs targeting the poor have expanded (Goldberg and Rosenthal, 2002; Sugiyama, 2011). This trend toward “pro-poor” social assistance has gained considerable strength in the early 2000s.

In many Global South countries, a dual welfare institutional structure has emerged, with older employment-based social insurance institutions existing alongside new or recently reformed social assistance programs targeting the poor. This duel system can be traced back to the origins of social protection in many Global South countries. Whereas the welfare states of the rich industrialized countries took significant steps toward equal access and expansion of institutions based around social insurance in the post-WWII era, social protection systems in middle-income and least developed countries often emerged alongside inward-looking economic development strategies (Haggard and Kaufman, 2020). The emphasis on protecting key economic sectors generally lead to generous social insurance institutions for the minority of formally employed workers in strategic, industrial, and public sectors. The poor in the Global South, which often worked in low-paying agricultural or informal sector jobs, were usually only eligible for limited benefits or ad hoc emergency aid. This situation forced the poor to rely on familial, tribal, or religious support networks for food or old age security. In sum, despite making up a vast majority of the population, the poor in low- and middle-income countries have typically been excluded from formal social protection systems or received very modest benefits.

**Figure 1:**
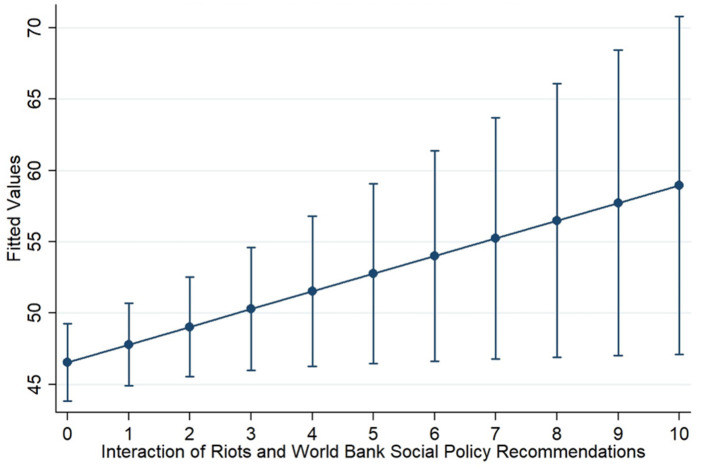
Average marginal effect with a 95% confidence interval.

As highlighted by Figure 1, this situation has recently begun to undergo significant changes as social assistance to the poor now plays a key role in the welfare institutions of EMEs. The comparison of averages changes in social assistance for different global regions^1^ indicates that Western countries have experienced a slight downward trend since 2004, while the Latin American and Asian countries show a clear upward trend in coverage rates. For the Eastern European countries, the gradual downward trend leading up to the global financial crisis appears to reverse after 2010. Thus, by the end of the sample period in 2013, coverage rates for three out of the four non-Western world regions approach that of the wealthy Western counties.

The recent rise of social assistance in the Global South is also highlighted by a variety of additional sources. Social assistance coverage reached the peak point of 54 percent in Indonesia, 24 percent in India, 61 percent in South Africa, 25 percent in Brazil, 44 percent in Turkey, and 59 percent in Mexico during the 2000s (World Bank, 2015). Along with overall coverage rates rivaling that of Global North countries, EME social assistance programs today reach an unprecedented total number of recipients. For example, the five largest social safety assistance programs in the world are provided by China, India, and Brazil to cover 486 million people, almost equaling the total European population (World Bank, 2015).

Individual social assistance programs in emerging markets also have the largest coverage in the world, including Bolsa Familia in Brazil with 29 percent coverage, Oportunidades in Mexico with 27 percent, Mahatma Gandhi in India with 16 percent, the free healthcare program in Indonesia with 31 percent, and the Child Support Grant in South Africa that covers 21 percent. In China, the Minimum Living Standard Guarantee Program (Dibao) has expanded to cover 75 million individuals and has become “one of the largest minimum income cash transfer schemes in the world” (World Bank, 2015). As a percentage of total government spending, social assistance spending increased 142 percent in Brazil, 266 percent in Turkey, and 281 percent in Mexico between 2000 and 2010 (Hall, 2006).

## Political and structural explanations of social assistance provision

While social safety nets have experienced a renewed interest in European countries (Bahle et al., 2011; Marx and Nelson, 2013), this trend is striking in the Global South, as well (Leisering, 2018). Yet, the contemporary global literature on social assistance programs is dominated by descriptive accounts, impact analyses, structural, institutional and ideational explanations, underestimating the effect of political—particularly contentious political—factors that influence the expansion of social assistance programs.

A recent systematic literature review conducted by Co-author 2 (2022) has illustrated that structural factors had the lion's share among determinants of social assistance. Structural factors mainly consist of economic conditions (opportunities or constraints), demography (aging, number of household members, marital status), labor market conditions (e.g., unemployment), human capital (educational status), state capacity, globalization (e.g., international trade) and poverty (Gupta et al., 2001; Barrientos and Santibáñez, 2009; DePolt et al., 2009; Fernandez, 2013; Fernandez and Jaime-Castillo, 2013; Bichir, 2016; Arza, 2018). Some of these structural explanations are rooted in a sizable literature on the mature welfare states of the original OECD countries (Esping-Andersen, 1990; Korpi and Palme, 1998; Hall and Soskice, 2001)—most of them are about non-OECD countries (Gough et al., 1997, 2004; Rudra, 2008 see also Haggard and Kaufman, 2008).

The institutionalist strand of scholarship examines social assistance programs by referring to the capacity and structure of welfare institutions. Scholars in this tradition analyze the extent to which the distribution of social assistance is centralized or localized in a government, the veto points in the political system, how social assistance policies (from caseworkers to welfare ministries) are structured, or how international institutions cooperate with the national governments (Bianculli et al., 2013; Smith and Urpelainen, 2017; Béland and Lecours, 2018; Jordan, 2018; Varjonen, 2020).

The third group in the publications on the determinants/causes of social addresses ideational factors—most importantly, the diffusion of ideas. They are interested in how professional norms and epistemic communities are created to shape social policy agenda, and how the bureaucrats and program implementers learn ideas that inform social policy-making in other geographies (Means and Smith, 1983; Sugiyama, 2011; Fenwick, 2013; Tomazini, 2019; Velázquez Leyer, 2020).

Political explanations of social assistance programs examine electoral competition, the political ideology of incumbent parties, share in cabinets or parliaments, social movements, protest events, ethnic conflicts, and governmental logic of discipline that influence the provision, distribution and expansion of social assistance programs. Our previous literature review (Yörük et al., 2022) illustrates that while studies on party politics constitute the majority of the publications on political determinants/causes, contentious politics is the least popular theme. In addition, publications on the social assistance programs in the Global South are more likely to analyze contentious political factors, whereas the publications in the Global North are more inclined toward party politics. Also, there is a shift in the focus of political analyses from the Global North to the Global South since the 1970s. By the late 2010s, political analyses have focused mainly on the Global South with a recent pickup in studies in the Global North.

Most publications that investigate the political determinants/causes of social assistance programs focus on party politics. The effect of political ideology on the social assistance policy-making processes is widely examined (Carnes and Mares, 2013; Aytaç, 2014; Göçmen, 2014; Lin, 2017; Silvestre, 2017; Borges, 2018). Within the limited scope of political analyses, contentious political factors are much less examined than party politics and governmentality. McAdam and Tarrow, 2013 (2013, p. 1) defines contentious politics as “episodic, public, collective interaction among makers of claims and their objects when: (a) at least one government is a claimant, an object of claims, or a party to the claims, and (b) the claims would, if realized, affect the interests of at least one of the claimants or objects of claims.” Therefore, it represents the dynamic interplay between the actors of non-institutionalized politics and the power holders such as “elites, opponents and the state” (1996, p. 874). In that sense, contentious politics covers a repertoire of contention, including social movements, protests, riots, conflicts, strikes, etc. (Tilly, 1993). When it comes to the limited volume of scholarship on contentious political determinants of social assistance, there are two different approaches. First, scholars argue that social assistance provisions come from negotiations and bargaining between grassroots actors and decision-makers (Garmany, 2017). Second, several other scholars show that governments use social assistance programs for the political containment of social unrest (Gutner, 2002; Seekings, 2007; Tabbush, 2010; Yörük, 2012; Calvo and Moscovich, 2017).

Other than the examples listed above, there are three clusters of studies which consider the welfare-contentious politics nexus, but they are all interested in the effect that welfare system change has on contentious politics. First, some scholars examine how social movements respond to *changing* welfare policies, rather than how welfare policies respond to *changing* social movements; such as the way in which social movements resist the retrenchment of welfare provisions, sometimes managing to force governments to step back (Pierson, 2001; Weiss, 2005; Poulantzas and Martin, 2008). Second, scholars have examined how social policies respond to “social welfare movements” which demand or organize around social welfare benefits (Agarwala, 2006; Vanhuysse, 2006; Mooney et al., 2009; Agarwala et al., 2015). But these studies tend to overlook the fact that a significant part of the effects of social movements are unintended, rather than intended consequences (Rucht, 1992; Giugni et al., 1998), including concession or repression (Tarrow, 1994; Della Porta, 2009). People may receive social assistance benefits while struggling for a social reform that is not related to social assistance. Lastly, a larger volume of scholarship, the so-called “welfare-terrorism nexus” literature, explores how increases in welfare provision reduces contentious political activism. In the rapidly expanding welfare-terrorism nexus literature, grassroots politics/social unrest/radicalism/insurgency has been considered as the dependent variable, while changes in welfare policies have been the independent variable. Our analysis looks at the opposite direction to examine how social assistance (dependent variable) changes as an intended and unintended consequence of violent civil unrest, even when spontaneous and unstructured, which does or does not demand policy changes.

In short, while structuralist approaches have provided important insights into the cross-national macro pressures which dictate policy actions toward certain welfare outcomes, in this narrative, the expansion of social assistance programs occurs almost as an automatic response to changing economic and demographic dynamics, while mostly ignoring the political processes that shape how these pressures are translated into actual social assistance policies. As such, except for a few recent case studies (Yörük, 2012; Li and Walker, 2016; Fallov and Blad, 2018; Yörük et al., 2019), the existing literature has generally under-examined the possibility that the contemporary expansion of social assistance is shaped by the political concerns of governments' political for containing social unrest. The studies which do exist point to the conclusion that social assistance is being used as a counter-insurgency and political control strategy in countries like China, Mexico, South Africa and Turkey. Still, there are no studies that examine whether this is a there is a global pattern in which governments use social assistance programs for counter-insurgency measures.

Our inquiry is inspired by the previously noted political approaches to the origins and transformation of welfare states. After the urban riots in the USA of the 1960s, scholars began to show theoretically and empirically how welfare was used for controlling, containing, and repressing (potentially) insurgent populations (Cowgill, 1974; Welch, 1975; Form, 1979; Gurr, 1980; Isaac and Kelly, 1981; Hicks and Swank, 1983; Jennings, 1983; Phillipson, 1983; Schram and Turbett, 1983; Trempe, 1983; Chamlin, 1989; Fording, 1997; Offe, 2018). Fox-Piven and Cloward's path-breaking work, *Regulating the Poor*, claimed that in order to maintain order and legitimacy, the modern state responded to racial and working-class riots by expanding poor relief programs. In times of social turmoil, relief systems expanded as a means of establishing control over the disorderly. However, when turmoil subsided, social assistance contracts (Cloward and Piven, 1971). Thus, this theory suggests that the independent variable for relief outcomes is not social need, but social disorder. Other scholars have put forward the conditions under which contentious politics, especially violence, leads to concessions, including the expansion of welfare policy (Button, 1978). The insights of this older generation of welfare scholars have recently been mostly ignored in explanations of welfare state changes in the global South and North, although the global and simultaneous presence of social unrest and the growth of ‘pro-poor' social assistance programs calls for the testing of such an hypothesis.

In this paper, we take up this aim by asking the following research question: Is greater social unrest related to a wider scope of social assistance coverage? Based on the theories outlined throughout this literature review, we expect riots to have a significant positive impact on social assistance coverage, all else being equal. The next section covers the data and methods used to statistically test this hypothesis.

## Data and methods

Our sample includes an unbalanced panel dataset of 54 original OECD and EME countries between the years 2002–2015, making for a total of 419 country-year observations. When referring to an emerging market economy, we are generally referring to countries which have many features of the economies of rich developed nations, such as high levels of economic growth and open markets, but industrialized later than many original OECD countries. Furthermore, EMEs typically lack one or more of the features found in many early industrializing countries, such as high GDP per capita, consistent socio-political stability, and high employment rates. To aid us in the identification of EMEs, we include countries listed on any one of three popularly applied lists^2^. We also include any relevant country which is listed by the World Bank as a middle-income or high-income during the sample period^3^. This leaves us with a sample of 17 original OECD and 37 EME^4^ countries.

The variables used in the main set of analyses are presented in Table 1. Our dependent variable is *social assistance coverage*, defined as the percentage of the population participating in social assistance programs (including direct and indirect beneficiaries)^5^. Coverage is measured as the number of individuals in the quintile who live in a household where at least one member receives the transfer, divided by the number of individuals in that quintile. Much of the data for the dependent variable comes from a recent World Bank initiative to comprehensively measure global social protection and labor market systems, titled the Atlas of Social Protection Indicators of Resilience and Equity (ASPIRE). Given the relatively early stage of the ASPIRE project, relying exclusively on World Bank social assistance coverage data still poses sample size challenges. To deal with this issue, we calculate a portion of data points for the dependent variable from the Luxembourg Income Study (LIS) using the same methodology as the World Bank, thus generating a complementary global dataset of social assistance coverage. Gaps in the sample are filled by taking the average of the next and previous years values.

**Table 1:** Summary statistics.

	**Count**	**Mean**	**SD**	**Min**	**Max**
Social assistance coverage (%Pop)	419	47.62846	21.58323	1.590127	92.74956
Social unrest index	419	1,046.142	2,879.466	0	26,500
Riots	419	0.8472554	2.338683	0	22
General strikes	419	0.202864	0.84683	0	8
Anti-gov demonstrations	419	1.26969	3.334797	0	30
FH political rights	419	5.847255	1.697327	1	7
Left government	419	0.3699284	0.4833622	0	1
GDP per capita, PPP (Logged)	419	9.7874	0.7609175	7.449151	11.11329
GDP growth	419	0.0472501	0.0437321	−0.1481638	0.2108831
Trade (%GDP)	419	83.9829	40.50576	21.12435	196.6091
Unemployment	419	7.28943	4.337414	0.49	27.47
Total population (Logged)	419	16.97693	1.602599	14.09162	21.02882
Old age dependency	419	18.34811	8.270525	5.142528	39.58342
Urban population (%Pop)	419	67.57986	17.78235	18.297	97.776
Original OECD country	419	0.3603819	0.4806852	0	1
**Left government**	**Freq**.		**Percent**		**Cum**.
0	677		67.16		67.16
1	331		32.84		100.00
Total	1,008		100.00		
**Original OECD country**	**Freq**.		**Percent**		**Cum**.
0	714		70.83		70.83
1	294		29.17		100.00
Total	1,008		100.00		

We define *civil unrest* as any form of mass civil disobedience in which participants become hostile toward authority, and authorities incur difficulties in maintaining public order. In order to measure civil unrest, we initially created an index comprising several key indicators (riots, anti-government demonstrations, and general strikes). As Table 2 demonstrates, of our civil unrest indicators, only riots presents a significant positive relationship with social assistance coverage. This result remains robust throughout different model specifications. As riots are the only form of civil unrest which necessarily involves violence, we take this finding to indicate that civil unrest alone may not be enough to induce policy-makers toward greater social assistance concessions on a scale which is detected by time-series statistical analysis. Rather, it may be necessary that civil unrest poses a serious threat to the state. We interpret this finding as a support for the argument that governments spend the most effort to deliver social policies that are expected to undermine the political activism of the most threatening social group. Compared to general strikes, for example, riots are more likely to be participated by “the poor,” i.e., the urban and rural populations, who mostly and irregularly work in the informal economy. Social assistance programs, by their design and implementation, target the poor, as opposed to social security programs, such as old age pensions or unemployment insurance, that typically cover formally employed peopled. One can argue that while social security programs are used to contain the political activism of formal workers, social assistance programs target the political activism of the poor, especially the violent ones, which can be statistically captured as the number of riots.

**Table 2:** Social assistance coverage and social unrest.

	**SAC**	**SAC2**
Social unrest index	0.0003	
	(1.34)	
Riots (total)		0.7626*
		(2.01)
Anti-Gov demonstrations (total)		−0.0910
		(−0.41)
General strikes (Total)		−1.0436
		(−1.60)
Constant	6.9226	18.7251
	(0.01)	(0.04)
		
Observations	419.00	419.00
Groups	54.00	54.00
R2	0.43	0.44

Prais-Winsten regression, panel corrected standard errors in parentheses.

^*^ p < 0.05, ^**^ p < 0.01, ^***^ p < 0.001.

As this whole process is likely to take more than a year after riots takes place, we test multiple lag lengths. Applying both Levin-Lin-Chu and BIC tests indicate the highest statistical validity for a two-year lag length, although models using alternative lag lengths yield similar results.

The measure of *riots* comes from the Cross-National Time Series (CNTS) domestic conflict event dataset, defined by the codebook (2017) as “any violent demonstration or clash of more than 100 citizens involving the use of physical force” (12). Events for this data are recorded through a comprehensive coding of international newspaper articles. With regards to sources, the CNTS user's manual notes that “while no bibliographic references are utilized in connection with these data, most are derived from *The New York Times*” (12). Demonstrating a causal relationship between social movements and welfare outcomes in a systematic way has often been difficult (Skocpol and Amenta, 1986; Giugni et al., 1998). This is partly because of a lack of data on social movements, as there are very few available sources on grassroots socio-political activity beyond labor strike statistics. The CNTS data offers the best cross-national measure for the entire period and sample of countries of interest by providing a reputable data source which has recently been widely used in articles published at major social sciences journals (Tenorio, 2014; Johnson and Thyne, 2018; Klein and Regan, 2018).

We also include several control variables in our models. First, *democracy* is often hypothesized to have a positive effect on social spending (Meltzer and Richard, 1981), which has been argued in studies focusing on both OECD and developing countries (Adsera and Boix, 2002; Rudra and Haggard, 2005; Acemoglu and Robinson, 2006). One assumption of this literature is that autocratic governments are less willing to redistribute wealth to the poor, especially if that means channeling state resources to members of rival groups (Jensen and Skaaning, 2015). Democracy is argued to give the poor an opportunity to form a strong constituency which can be mobilized by political parties (most likely with left-leaning political-economic ideologies) and interest organizations, thus increasing the pressures for redistribution. For instance, it has been hypothesized that the expansion of “pro-poor” welfare policies in the Global South has partly been facilitated by the “third-wave” democratic transitions, which were expected to make policy-makers more responsive to the needs of the “median” poor voters (Mares and Carnes, 2009; Haggard and Kaufman, 2020). We, therefore, include *democracy* as a control variable in all of our models. As one of the most widely used measures of democracy, we include Freedom House's (2016) political rights scores^6^. Along with democracy, we control for left ruling party governments (Database of Political Institutions, 2015), as ideologically left parties are expected to favor redistributive policies.

We also control for several macroeconomic influences. Most importantly among these, we include *GDP per capita*^7^ World Bank (2015) as our measure of wealth. Wealth should have a significant relationship with social assistance, all else being equal, as a greater share of resources can be allocated to social assistance programs. Furthermore, wealthy countries tend to have greater institutional capacity to reform and deliver welfare. Additionally, we include *GDP growth*, thus controlling for the possibility that variation in the dependent variable is actually caused by differences in growth rates. An extensive literature has now been written on the relationship between economic globalization and welfare, with opinions still split on whether globalization leads to welfare expansion or retraction for the poor (Adsera and Boix, 2002; Rudra, 2008). The literature on OECD welfare states suggests that greater economic openness is expected to generate pressures for social protection, as well as relatively greater shares of investment in general skills. On the other hand, research on the Global South tends to suggest that a “race to the bottom” is taking place^8^ (Kaufman and Segura-Ubiergo, 2001; Rudra, 2002; Avelino et al., 2005; Wibbels, 2006), the logic being that developing countries lack the domestic factors which would mediate the deleterious effects of globalization found in the original OECD countries (for instance strong democratic institutions) (Rudra, 2008; Haggard and Kaufman, 2020). Several models therefore control for *trade* as a proxy for globalization^9^ (IMF, 2015). Finally, given the previously discussed effects of *unemployment* on both unrest and welfare, we control for unemployment in all of our models^10^ (World Bank, 2015). This helps us because social unrest (Riots) could be indicators of social crisis, therefore, this model helps us to differentiate between the Riot (as a social outburst) from the structural crisis in itself. This would suggest that a driving force of the social assistance coverage is social unrest per se, net of the structural crisis (e.g., unemployment, recession, etc.). Structural factors, such as economic crisis, are translated into social assistance policymaking through the mediation of contentious political factors. Such structural factors set up the constraints within which contentious political factors determine the eventual trajectory of welfare changes. These mechanisms involve conflicts among competing political actors, mainstream and non-mainstream. The key factors determining the transformation of social assistance provision is therefore the threat of social unrest.

We also include a variety of demographic control variables. *Population* size (World Bank, 2015) is included to take into account the popular argument in the welfare state literature that larger countries face greater difficulty expanding social assistance coverage than smaller countries. This is in part because countries with a greater population sizes must reach a larger number of people and/or because small countries should have an easier time establishing solidarity on a national level in order to organize parties or unions that can influence national politics (Katznelson, 1981). Smaller countries are also expected to be more exposed to global market risks, thus workers should demand greater protection. We include a measure of *urban* population size (World Bank, 2015) in order to control for unrest caused by the urban poor, as well as the expected “pro-welfare” effects of urbanization discussed in the literature review. Lastly, we control for *old age dependency* (World Bank, 2015), as an older population should in theory drive up the demand for greater social assistance coverage. On the other hand, the majority of EMEs in the sample have relatively young populations. As noted previously, the group of EMEs in our sample have shown the greatest amount of recent growth in social assistance. It might also be the case that youth populations are more restless and likely to partake in violent demonstrations, thus requiring greater appeasement through welfare concessions.

Finally, several models take into consideration the effect of the diffusion of ideas on social assistance coverage. As an indicator of donor diffusion of policy ideas does not currently exist, we create a new measure based on the number of *World Bank social policy recommendations* (WBSPR). This measure is informed by a survey of a total of 447 World Bank social policy-related documents and reports^11^. The core of the WBSPR measure counts the number of World Bank social policy recommendations given to a specific country in a given country-year^12^. This score is then added to the number of relevant regional reports. Finally, the World Bank occasionally publishes several highly influential policy documents which apply to every recipient country for the year in which the document was published. Thus, a single point is also added to every country-year in which an influential multi-country document is published.

Given the early stage of the data on social assistance coverage, we are inevitably faced with missing data. In such a context, Beck and Katz (1995) do not suggest calculating standard errors using the generalized least squares estimation technique, especially when the number of cases exceeds the length of the time-series. Post-estimation also reveals the presence of serial autocorrelation and heteroskedastic standard errors^13^. Thus, we apply a Prais-Winsten regression analysis with panel-corrected standard-errors (PCSEs) and a common AR(1) process, as advocated by Beck and Katz (1995). To control for potentially exogenous time trends which could produce spurious relationships, we include a year-count variable (omitted from output tables). All control variables are lagged by 1 year, unless stated otherwise, as we expect their effects to take place in the short-term but not instantly. All models apply pairwise list deletion. The equation for the model is as follows:

sac_it − 1_ = α + β1riots_it − 1, 2_ + Σ(β_k_controls)_it − 1_ + ε_*t*_

As noted previously, in the above model both one- and two-year lag lengths are applied for the riot's variable. Theoretically, we expect that it will take more than a year before the effects of riots are translated into actual social assistance coverage changes. Insurgent populations may be repressed through malevolent means (i.e., police crackdowns, mass incarcerations, etc.) before policymakers attempt to prevent further unrest through benevolent welfare concessions. Governments may use a variety of options available to deal with domestic social unrest. On the one hand, the “malevolent” social control hypothesis would argue that policy-makers increase public order spending and the size of the police force in order to repress ongoing unrest. On the other hand, the “benevolent” social control hypothesis would argue that policy-makers increase social protection spending in order to co-opt and contain those members of insurgent populations. Governments may choose some combination of the first two options, such as repressing unrest in the short-term and then extending social protection in order to reduce the likelihood of the recurrence of violence. Which spatial and temporal configurations of welfare and repression are more effective can be the subject of further analyses. Here, it suffices to say that statistically, a two-year lag length is shown to provide slightly more robust results than a single lag on the riots variable.

## Results

Our quest for finding a general explanation for contemporary global social assistance provision has led to a number of empirical observations. The results for the first set of models can be found in Table 3. Across all five models riots are positive and highly significant. This result remains robust across a variety of different model specifications. We interpret this finding as evidence in favor of the argument that policymakers extend social assistance coverage in order to contain further unrest. The Models in Table 3 also generally indicate an important influence of *structuralist* factors, such as trade and age.

**Table 3:** Social assistance coverage and violent civil unrest.

	**Model**	**Model**	**Model**	**Model**	**Model**
	**I**	**II**	**III**	**IV**	**V**
Riots (total)	0.82***	0.82***	0.84***	0.84***	0.84***
	(0.19)	(0.21)	(0.21)	(0.21)	(0.21)
FH political rights	0.11	0.37	0.37	0.42	0.35
	(0.71)	(0.63)	(0.64)	(0.64)	(0.65)
GDP per capita, PPP (logged)	8.37***	14.20***	14.15***	14.04***	13.06***
	(1.78)	(1.65)	(1.70)	(1.56)	(2.14)
GDP growth	−13.46*	−17.50**	−16.91**	−17.90**	−17.34**
	(5.69)	(6.00)	(5.89)	(6.20)	(6.05)
Trade (%GDP)	0.11**	0.13***	0.13***	0.14***	0.13***
	(0.04)	(0.04)	(0.04)	(0.04)	(0.04)
Total population (Logged)	−3.12***	−3.07***	−3.14***	−3.05***	−3.04***
	(0.94)	(0.88)	(0.91)	(0.89)	(0.92)
Old age dependency		−0.80***	−0.79***	−0.79***	−0.78***
		(0.20)	(0.21)	(0.18)	(0.19)
Left government			1.37	1.43	1.33
			(1.09)	(1.13)	(1.07)
Unemployment				−0.06	−0.06
				(0.22)	(0.22)
Urban population (%Pop)					0.07
					(0.10)
Constant	988.07*	1,240.75*	1,217.57*	1,246.92**	1,203.04*
	(498.16)	(481.83)	(482.94)	(483.68)	(491.70)
Observations	419.00	419.00	419.00	419.00	419.00
Groups	54.00	54.00	54.00	54.00	54.00
R2	0.55	0.57	0.57	0.57	0.57

Prais-Winsten regression, panel corrected standard errors in parentheses.

^*^ p < 0.05, ^**^ p < 0.01, ^***^ p < 0.001.

Starting with Model 1, all relationships are in the expected direction, with the exception of democracy which remains insignificant. Macroeconomic effects, such as wealth and trade, show a strong positive relationship with social assistance coverage. The positive relationship between trade and coverage is interesting, in that it sheds some doubt on the proposition that a “race to the [neoliberal] bottom” is taking place in social services due to globalization. However, since the literature also expects globalization to effect countries differently based on development, this relationship is revisited in later models which disaggregate the sample between OECD and EME countries. Model 1 also indicates a significant negative relationship of population size, supporting the case that states with smaller populations, in general, are more prone to extending social assistance coverage.

The inclusion of old age dependency in Model 2 has an intriguing negative relationship with riots. This result is contrary to the expectation that large old age populations drive up the demand for social assistance coverage. The negative finding for old age could suggest that countries with comparatively younger populations, such as many of the EMEs, have a positive correlation with social assistance coverage. A plausible explanation for this could be the tendency of younger populations to be more prone toward social unrest, thus instigating the need for greater social containment. However, without data on the age composition of the rioting population, drawing this conclusion is still premature.

The inclusion of majority left party governments' in Model 3 is positive but insignificant, albeit by a slim margin. Considering that social assistance is far from residual in recent global welfare institutional structures, we suggest that the explanation for the political motivations underpinning social assistance expansion can be found in the writings of the mid-20th century welfare state scholars who argued that social assistance provision is used as a social containment strategy during periods of turmoil (Cloward and Piven, 1971). From this perspective, the coincidence of strong social assistance and social insurance institutions in EMEs is a logical outgrowth of the need for policymakers to address two different sources of political pressure—the containment of social unrest and vote maximization. Furthermore, the need to extend social assistance in order to contain social unrest should cross-cut party lines, thus being employed by both right and left parties.

Interestingly, *democracy* has no significant relationship with social assistance coverage, a result which remains robust across different model specifications. In line with the above discussion, this could be due to the political pressures faced by politicians in a variety of different regime types. This result has potentially important implications for the literature which tends to examine democracy as a key political explanation of welfare expansion. Although the research on democracy and welfare in original OECD countries is based on strong empirical foundations, an increasing number of studies have begun to question the extent to which these theories apply to the Global South (Dietrich and Bernhard, 2016). For instance, it remains a mystery as to why the rapid adoption of social assistance programs in EMEs would occur nearly two decades after the “third wave” democratic transitions and across a diversity of regime types. Furthermore, in Latin America the evidence suggests that the “third wave” of democracy actually coincided with rising inequality (Huber and Bogliaccini, 2010). Ross (2006) proposes that evidence of a positive relationship between democracy and improved welfare outcomes is actually a result of sample bias toward wealthy democracies. This result therefore deserves further investigation which looks specifically at the link between democracy and social assistance. Models 4 and 5 test for *unemployment* and *urbanization*. Contrary to our expectations, neither one of these variables show a statistically significant effect, nor do they appear to influence the direction or magnitude of the results for riots. This raises doubts about theories which expect economic uncertainty associated with rising unemployment to be an important structural pressure on policymakers to expand social assistance.

As discussed previously, the literature on welfare has often focused on original OECD countries at the expense of EMEs or least developed countries. This dichotomy implicitly suggests that the poorer non-OECD countries do not have the characteristics necessary to be considered welfare states. Given the growing abundance of welfare institutions in EMEs, a strong argument against this assumption can be made. However, differences in levels of wealth should have an important conditioning effect on how some factors influence welfare institutional structures. For instance, EMEs are generally faced with greater political and social instability, while also having overall less resources to either suppress this unrest or to benevolently extend welfare to contain it. We thus expand our analysis in Table 4 by directly testing the differences between EME and OECD countries.

**Table 4:** OECD vs. EME.

	**Model**	**Model**	**Model**	**Model**
	**VI**	**VII**	**VIII**	**IX**
Riots (total)	0.83***	0.89	0.85***	0.81***
	(0.21)	(0.68)	(0.26)	(0.21)
FH political rights	0.31	2.18	0.32	0.36
	(0.66)	(4.10)	(0.70)	(0.58)
GDP per capita, PPP (logged)	11.05**	32.74**	8.73**	17.22***
	(3.46)	(12.35)	(3.17)	(3.50)
GDP growth	−16.56**	−23.18*	−17.44*	−17.37**
	(6.17)	(10.54)	(6.98)	(6.23)
Trade (%GDP)	0.13***	0.09*	0.17***	0.10**
	(0.04)	(0.04)	(0.05)	(0.04)
Total population (logged)	−3.37***	−5.75*	−2.46*	−5.19***
	(1.02)	(2.24)	(1.09)	(0.95)
Old age dependency	−0.86***	−0.33	−0.75**	−0.45
	(0.19)	(0.52)	(0.27)	(0.28)
Unemployment	−0.07	0.07	0.09	
	(0.23)	(0.44)	(0.24)	
Urban population (%Pop)	0.09	−0.00	0.14	
	(0.10)	(0.12)	(0.11)	
Original OECD country	4.38			
	(3.95)			
Eastern Europe				−1.87
				(5.18)
Latin America				6.87
				(5.23)
Asia				20.85**
				(7.86)
North Africa and Middle East				7.47
				(11.27)
Sub-Saharan Africa				8.50
				(10.19)
Constant	999.04	2,041.91	1,071.60	1,553.19*
	(548.52)	(1,256.71)	(627.45)	(611.48)
Observations	419.00	151.00	268.00	419.00
Groups	54.00	17.00	37.00	54.00
R2	0.57	0.76	0.49	0.57

Prais-Winsten regression, panel corrected standard errors in parentheses.

^*^ p < 0.05, ^**^ p < 0.01, ^***^ p < 0.001.

Model 6 applies the full sample of countries but includes a dummy variable for OECD (1) vs. EME countries (0). This model indicates there no clear relationship in favor of either rich OECD countries or EMEs. Models 7 and 8 take this analysis a step further by splitting the sample of cases between OECDs and EMEs. This comparison is highly telling in that the positive influence of riots in the full sample appears to be partly driven by the sample of EMEs in Model 8. It should, however, be noted that caution is required when drawing conclusions based on the splitting of the sample due to a number of statistical biases which are introduced. Yet, with that in mind, splitting the sample into two different groups does provide some evidence to suggest that the focus on Western countries, which has dominated the literature on welfare states, may be partly to blame for the underappreciation of contentious political factors in the welfare literature. In other words, there is some evidence to suggest that violent civil unrest is a more potent driver of social assistance in EMEs. Thus, a sample bias has led researchers to undervalue the role that unrest plays in social assistance policy formation in contemporary research and to overvalue the significance of *structural* factors. Evidence of this can also be seen in Model 9, which includes dichotomous variables for each EME world region included in the dataset. In line with our previous results, all the EME regions except for Eastern Europe are positive. Moreover, while Asia is significant, Latin America is insignificant by a only a slim margin.

Table 5 provides robustness checks by testing alternative model specifications. Beginning with models 10 and 11, there are reasons to believe that international financial institutions (IFIs), such as the World Bank, may be playing a mediating role in the relationship between contentious politics and social spending. While the World Bank claims that many new social policies (such as social pensions and conditional cash transfers) in borrowing countries are a result of technocratic (read: non-political) imposition (Brooks and Manza, 2006; Radin, 2008), several recent studies have shown that political objectives have unsurprisingly played a critical role in the content of World Bank recommendations, including the prevention and containment of social unrest (Van Gils and Yörük, 2017). These studies have shown that World Bank social policy recommendations are not solely based on technocratic concerns over poverty alleviation and development but are fueled by the Bank's own concerns over political stability. Thus, these observations have led us to question whether the World Bank's recommendations are taken into consideration more seriously by borrowing countries if they are challenged by social unrest.

**Table 5:** Alternative model specifications.

	**Model X**	**Model XI**	**Model XII**	**Model XIII**	**Model XIV**
Riots (total)	0.85***	−0.24			
	(0.19)	(0.56)			
Ethnic fractionalization			0.13*		
			(0.06)		
CSO anti-system movements				0.42	
				(1.06)	
FH political rights	0.33	0.35	0.38	0.22	0.36
	(0.64)	(0.63)	(0.65)	(0.65)	(0.67)
GDP per capita, PPP (logged)	10.70***	10.58***	10.88***	10.86***	11.02***
	(2.14)	(2.05)	(2.25)	(2.45)	(2.27)
GDP growth	−15.35**	−15.22**	−14.78*	−14.38**	−15.45**
	(5.80)	(5.79)	(5.81)	(5.52)	(5.68)
Trade (%GDP)	0.13***	0.13***	0.13***	0.11**	0.13***
	(0.04)	(0.04)	(0.04)	(0.04)	(0.04)
Total population (logged)	−4.11***	−4.05***	−4.08***	−4.10***	−3.82***
	(0.94)	(0.93)	(0.97)	(1.06)	(0.99)
Old age dependency	−1.01***	−1.01***	−1.04***	−0.82***	−0.99***
	(0.22)	(0.21)	(0.22)	(0.20)	(0.22)
WB social policy recommendation (dummy)	−10.72***	−11.19***	−10.61***	−10.65**	−10.25**
	(2.98)	(2.79)	(3.12)	(3.67)	(3.24)
Riots x WBSP		1.24*			
		(0.60)			
Constant	796.90	800.57	769.10	683.72	678.17
	(517.94)	(501.57)	(518.93)	(487.92)	(507.92)
Observations	419.00	419.00	419.00	419.00	419.00
Groups	54.00	54.00	54.00	54.00	54.00
R2	0.57	0.58	0.56	0.56	0.56

Prais-Winsten regression, panel corrected standard errors in parentheses.

^*^ p < 0.05, ^**^ p < 0.01, ^***^ p < 0.001.

As an indicator of donor diffusion of policy ideas does not currently exist, we create a new measure based on the number of *World Bank social policy recommendations* (WBSPR). This measure is informed by a survey of a total of 447 World Bank social policy related documents and reports^14^. The core of the WBSPR measure counts the number of World Bank social policy recommendations given to a specific country in a given country-year^15^. This score is then added to the number of relevant regional reports. Finally, the World Bank occasionally publishes several highly influential policy documents which apply to every recipient country for the year in which the document was published. Thus, a single point is also added to every country-year in which an influential multi-country document is published. The results for the WBSPR variable in model 10 point to an unexpected negative effect of the World Bank on social assistance coverage. However, in model 11 we find that the effects of the interaction between WBSPR and riots is positive and significant. Thus, there is some evidence of a relationship between World Bank social policy interventions and government decisions to spread social assistance coverage as a reaction to social unrest. In other words, governments take into account World Bank social policy recommendations to the extent that they are challenged by violent social unrest. This is an important finding that contributes in the expanding literature on international institutions influence on public policies. We encourage future studies to take up this research by examining the micro-foundations of the relationship between IFI influence and social assistance.

Model 12 includes *ethnic fractionalization* (Fearon, 2003) in place of riots. A commonly held assumption is that states with higher levels of ethnic fractionalization should have greater intercommunal conflicts and ethnic agitations which can contribute to civil unrest (Fearon, 2003; Alesina et al., 2004). The conventional wisdom, however, is that greater civil unrest has a detrimental influence on welfare outcomes. As can be seen in model 12, ethnic fractionalization actually has a significant positive relationship with social assistance coverage, which raises doubts about the common assumption that ethnically fractionalized societies are less willing to redistribute wealth.

Ethnic fractionalization is expected to have a negative relationship with social assistance under the assumption that wealthier members of ethnically homogenous societies will have a more favorable attitude toward social redistribution if they feel a stronger kinship to other members of society (Luttmer, 2001; Habyarimana et al., 2007). However, the empirical evidence supporting this conclusion is mainly derived from research on the advanced Western welfare states, with the United States serving as the key case of an ethnically fractionalized society. It is certainly possible that this same logic applies to welfare in developing countries, although more research is needed. Yet, it is often taken for granted in the political economy literature that elites in ethnically fractionalized developing countries will favor redistributive policies toward their own ‘insider' ethnic groups (Alesina et al., 2004; Jensen and Skaaning, 2015). This assumption ignores a key role of public social assistance spending in ethnically fractionalized societies which revolves around containing ethnic threats emanating from minority “outsiders”. Examples include the Green Card free healthcare program used against the Kurdish unrest in Turkey (Yörük and Gençer, 2020), PROSPERA against Zapatistas in Mexico (Yörük et al., 2019). From this perspective, the political origins of redistributive politics may have less to do with attitudes about opposing ethnic groups by middle class voters (Mares and Carnes, 2009), but rather represents a form of security measure against ethnic political instability. In such a context, it is possible to connect greater ethnic fractionalization to increases in social assistance provision.

Finally, Model 13 applies the Varieties of Democracy (V-Dem) (volume 9, 2019) civil-society organization anti-system movement variable in place of riots. This is an expert-coded variable that defines anti-system opposition movements as “any movement—peaceful or armed—that is… organized in opposition to the current political system” (V-Dem Codebook 182). While this anti-system movement variable provides inconclusive results, we remain confident with our original significant findings that utilize CNTS riots data as the key measure of social unrest as it does not rely on expert coding.

## Discussions and conclusions

Since the 1990s, there has been a global expansion of social assistance programs and this expansion is particularly striking in emerging markets economies. There is also a parallel surge in the literature on the causes and dynamics of this social assistance expansion. As we have pointed out, this literature has thus far suffered from the predominance of structuralist perspectives, which emphasize demographic and economic factors. These studies tend to present welfare expansion as an almost automatic response to a number of factors, such as aging, poverty, globalization, deindustrialization, informality, and the financial constraints of traditional welfare programs. This structuralist paradigm has overshadowed politics-centered explorations of social assistance provision, despite the fact that such political analyses, which focused on civil unrest, political competition, etc, enriched the literature on the mid-20th century development of social welfare—the so-called “golden age of capitalism”. The existing political explanations presented in the literature have thus far emphasized the effects of democracy, which, according to scholars, is expected to give political leverage and bargaining power to the poor and hence lead to higher social assistance. Nevertheless, there are very few systematic and empirical analyses of the ways in which social unrest, civil disorder, or political instability in general urge policymakers to expand social assistance programs as measures of public security.

Our analyses aimed at filling this gap in the literature. We began our analysis by inquiring about the relationship between violent civil unrest and social assistance. Our argument can be broadly summarized as: governments seek to extend social assistance to politicized groups that pose a threat to the state, thus using social assistance as a means of social control. The results of our analysis indicate a positive relationship between social assistance coverage and violent civil unrest, which is represented by the incidences of riots. The implications of this finding is that social assistance policies are adopted and extended by policy-makers, at least partially, as a securitization strategy to contain riots. Moreover, we suggest that the securitization of social assistance policies depends on an understanding by policymakers that contentious groups may transform grievances into further political activism. Therefore, alleviating these grievances through social assistance is seen as an “instrument”, rather than an end in itself, to undermine the conditions of this radicalization. We have also shown that ethnic fractionalization, which has been considered in previous studies as detrimental to welfare provision, has a strong positive relationship with social assistance coverage. We therefore suggest that our results may be partially related to government efforts to stabilize ethnic grievances by extending social assistance to outsider ethnic groups.

Another counter-intuitive finding of our analysis is that democracy has no significant relationship with social assistance coverage. Thus, we raise doubts about democracy serving as the driving political force behind the recent expansion of social assistance. We also find indications that the relationship between social assistance and violent civil unrest is more prominent in emerging market economies, as opposed to original OECD countries. In this sense, our findings add a global dimension to a flourishing literature on the political dynamics of social assistance in emerging markets such as China, Mexico, South Africa and Turkey, by giving a more structured picture to an already emerging pattern of counter-insurgency use of anti-poverty programs. This is important, as it reveals the salience of the civil unrest in the Global South, which may help explain the underuse of contentious politics-based explanations in the vast contemporary research for the Global North. Finally, our results indicate that World Bank social policy recommendations have a positive effect on social assistance coverage only when there are riots. This implies that governments take World Bank recommendations seriously when they are challenged by violent social unrest and when they see World Bank recommendations as a remedy for political instability. This finding has important implications regarding the relationship among grassroots groups, governments and transnational organizations.

But, what makes social assistance an instrument of containment against violent civil unrest? We now would like to discuss some hypotheses about the potential mechanisms in which riots lead to greater social assistance. Several authors have noticed how political unrest is linked to the social welfare situation in a country, and how social programs are regarded by state elites as a tool to contain this unrest (Paxson, 2002; Burgoon, 2006). As an underlying theme of this literature, policymakers believe that poverty and inequality stimulate feelings of injustice and thus can stimulate the mobilizing capacity of insurgents, fueling civil and ethnic conflict (Gurr, 1980; Auvinen and Nafziger, 1999; Paxson, 2002; Fearon and Laitin, 2003; Li and Schaub, 2004; Chen, 2009). Therefore, it is argued, governments see those measures targeting poverty as useful tools to reduce political radicalism that emanate from the poor.

Many scholars have argued that the poor today have become a major source of grassroots political power. In *Planet of Slums*, Mike Davis has called global slums the new center of socio-political conflict. For Davis, the debt crisis of the 1970s, the subsequent structural adjustment policies led by the IMF and the World Bank, processes of agricultural deregulation as well as de-peasantization, and finally expanding civil/ethnic wars and conflicts in regional peripheries have created push instead of pull factors, which led to the rapid exodus of rural populations into the cities. In cities that are unable to absorb this migration *via* formal networks of employment and accommodation, large slum areas, as places of “a surplus population”, have appeared (Davis, 2004).

Political struggles of the poor can become manifest in growing ethnic, religious, and gender-based inequalities, potentially producing radical threats to political stability. According to Arrighi (2009), the surplus population of the contemporary world is increasingly acquiring an *ethnic characteristic on the national level* and *national characteristics on the global level* and creating increasing political instability (Arrighi, 2009). Wacquant (2008) has emphasized that the contemporary rise of ethnic urban marginality tend to coalesce urban inequalities, ethnic cleavages, and political unrest. The uprisings in Egypt, Tunisia, Haiti, Greece, and Turkey are likely to show that poor people of the world are not passive victims of neo-liberalism, but rather they emerge as important political threats capable of paralyzing existing political regimes. Religious movements such as Islamism and Pentecostal Christianity as the “religions of the informal periphery,” ethnic militias, street gangs, and revolutionary social movements emerge as the agencies able to mobilize the global residuum (Davis, 2004).

This is of course not to say that all areas afflicted with growing poor populations will automatically be disposed toward social unrest. In fact, in Latin America, rising inequality accompanied the longest period of stable democracy ever. However, the Latin American cases also highlight the initiatives taken by politicians to curb the grievances of poor groups before they become radicalized. Many of the slums in this region are bastions of clientelist politics (or scenarios in which drug lords placate residents with resources of various kinds), leading to political quiescence rather than unrest. Yet, in addition to these formal or informal populist mobilization strategies, social assistance programs have also been created or their coverage extended as a response to the growing political power of the poor as source of threat for governments in a strategy for their political containment.

Burgoon argues that “social welfare policies may reduce international and domestic terrorism” by “diminishing grievances underlying extremist action” (Burgoon, 2006, p. 176). Burgoon asserts that welfare efforts reduce poverty, inequality, and socio-economic insecurity, and thus they diminish incentives to commit, support, or tolerate terrorism. Social policy also develops “citizens' perceived economic security” (Burgoon, 2006, p. 183) and creates ideological attraction for government policies as opposed to radical groups' imperfect substitute for economic security and equality. Social policy can also happen to reduce “horizontal inequality”, i.e., economic inequality that coincides with ethnic or religious divisions in a society, by leveling out income differentials between different social groups and thus lowering the likelihood of terrorism (Stewart, 2004). Taydas and Peksen (2012) also find a negative correlation between government spending on social security and the exposure to conflicts, concluding that social welfare spending is a useful governmental tool for reducing civil conflicts with a dual effect. On the one hand, favorable social policy measures can assimilate oppositional movements and bring about legitimacy for governments by decreasing people's grievances. On the other hand, social spending by the government can also reduce the chances of political insurgency by subverting (part of) the motives (Taydas and Peksen, 2012, p. 277–8; Chenoweth, 2007). Other scholars believe that social safety net programs may contribute to political stability, especially when unemployment levels are high. Empirically assessing the influence of social policies on home-grown terrorist activities in fifteen Western European countries between 1980 and 2003, Meierrieks and Krieger (2009) argue that social policies such as health, unemployment benefits, and active labor market programs indirectly reduce terrorist activities by ameliorating poor short-run and long-run socioeconomic conditions. We contend that similar observations have likely urged policymakers to expand social assistance as a soft-technique to deal with grassroots threats.

We argue that demographic and economic exigencies do not automatically lead to changes in welfare policies. Rather, socio-structural factors are translated into social welfare programs through political conflict and struggles. Specifically, Cloward and Piven (1971) thesis may help us to understand the recent expansion of social assistance programs targeting the poor. Over the last two to three decades, there have been remarkable changes in grassroots politics at the global level, which have likely shaped welfare reforms. There is a new group of the world's most disadvantaged sectors, people of global slums, who have acquired wide political significance as a source of political threat over the last couple of decades. Fox-Piven and Cloward's claim that mass turbulence by poor people's movements produces social welfare concessions needs to be examined in light of the contentious politics emanating from these slums. Today, while social assistance is becoming a dominant trend in the Global South, Fox-Piven and Cloward's thesis needs even greater revisiting and consideration at a global level.

## Data availability statement

The original contributions presented in the study are included in the article/Supplementary material, further inquiries can be directed to the corresponding author. We have used our novel dataset of welfare generosity and welfare effort, the Global Welfare Dataset (https://glow.ku.edu.tr/), which is an outcome of the ERC- funded Emerging Welfare Project (emw.ku.edu.tr). As a contribution to the global welfare scholarship, the GLOW Dataset introduced social assistance-related variables for all sample countries from Global North and South 50520.

## Author contributions

All authors listed have made a substantial, direct, and intellectual contribution to the work and approved it for publication.

## Conflict of interest

The authors declare that the research was conducted in the absence of any commercial or financial relationships that could be construed as a potential conflict of interest.

## Publisher's note

All claims expressed in this article are solely those of the authors and do not necessarily represent those of their affiliated organizations, or those of the publisher, the editors and the reviewers. Any product that may be evaluated in this article, or claim that may be made by its manufacturer, is not guaranteed or endorsed by the publisher.

## Footnotes

^1^The data presented in Figure 1 comes from the World Bank Atlas of Social Protection Indicators of Resilience and Equity (ASPIRE) project or are author-coded using the World Bank methodology with data from the Luxembourg Income Study (LIS).

^2^These include the International Monetary Fund (IMF), Banco Bilbao Vizcaya Argentaria (BBVA), and the Emerging Market Bond Index Global (EMBI Global) by J.P. Morgan.

^3^Countries with a population less than 500,000 are also excluded from the sample.

^4^For a full list of countries see appendix.

^5^By social assistance programs, we refer to cash transfers and near-cash (means tested or not), conditional cash transfers, social pensions, in-kind transfers, school supplies, public works, food for work programmes and fee waivers or targeted subsidies for health care, schooling, utilities or transport (World Bank, 2015).

^6^Freedom House Political Rights Score are expert coded scores assigned each year through evaluation by in-house and external analysts and expert advisers from the academic, think tank, and human rights communities based on the relative freedom of a country's electoral process, political pluralism, participation, and the functioning of the government. Political Rights Score is an indexed of a wide range of factors that are defined in the Freedom in the World Research Methodology Handbook (https://freedomhouse.org/reports/freedom-world/freedom-world-research-methodology). We reverse coded from political rights as 1 (least democratic) to 7 (most democratic).

^7^Current international dollars, adjusted for purchasing power parity.

^8^The “race to the bottom” hypothesis can be generally summarized as stating that high universal welfare standards are incompatible with market competition (Mishra, 1996; Wibbels, 2006).

^9^Measured as exports plus imports as a percentage of GDP.

^10^Measured as the unemployed population as a percentage of the total labor force.

^11^These include Country Focus reports, Economic and Sector work reports, Project Documents and documents from the Publications and Research department, including working papers. These are thus analytical as well as operational reports.

^12^To create this measure, we build on a previous dataset which followed a two-stage keyword search method (Van Gils and Yörük, 2017). In the first stage, documents on social policies are identified from the World Bank's online archive. In the second stage, these documents are searched for 24 specific keywords that are likely to indicate the World Bank's interest in political objectives. NVivo 10 is used to code all relevant documents.

^13^Post-estimation for this analysis includes breusch-pagan tests for heteroskedasticity and the Levin-Lin-Chu unit-root test.

^14^These include Country Focus reports, Economic and Sector work reports, Project Documents and documents from the Publications and Research department, including working papers. These are thus analytical as well as operational reports.

^15^To create this measure, we build on a previous dataset which followed a two-stage keyword search method (Van Gils and Yörük, 2017). In the first stage, documents on social policies are identified from the World Bank's online archive. In the second stage, these documents are searched for 24 specific keywords that are likely to indicate the World Bank's interest in political objectives. NVivo 10 is used to code all relevant documents.
