# Supplementary material for: The squeaky wheel gets the grease: Violent civil unrest and global social assistance provision
Source: Front Sociol. 2022 Oct 5;7:891267. doi: 10.3389/fsoc.2022.891267 (PMC9580367; doi:10.3389/fsoc.2022.891267)
Supplement: Supplementary file 1 [file Table_1.docx]

| Table 6: Countries | |
| --- | --- |
| Albania | Japan |
| Argentina | Jordan |
| Austria | Kazakhstan |
| Bangladesh | Latvia |
| Belarus | Mexico |
| Belgium | Netherlands |
| Brazil | Nigeria |
| Bulgaria | Norway |
| Chile | Pakistan |
| China | Panama |
| Colombia | Paraguay |
| Costa Rica | Peru |
| Croatia | Philippines |
| Czech Republic | Poland |
| Denmark | Portugal |
| Dominican Republic | South Africa |
| Ecuador | Spain |
| Estonia | Sri Lanka |
| Finland | Sweden |
| France | Switzerland |
| Germany | Thailand |
| Greece | Turkey |
| Hungary | Ukraine |
| India | United Kingdom |
| Indonesia | Uruguay |
| Ireland | Vietnam |
| Italy |  |
