# Supplementary material for: The squeaky wheel gets the grease: Violent civil unrest and global social assistance provision
Source: Front Sociol. 2022 Oct 5;7:891267. doi: 10.3389/fsoc.2022.891267 (PMC9580367; doi:10.3389/fsoc.2022.891267)
Supplement: Supplementary file 2 [file Table_2.docx]

| Table 7: Years |
| --- |
| 2002 |
| 2003 |
| 2004 |
| 2005 |
| 2006 |
| 2007 |
| 2008 |
| 2009 |
| 2010 |
| 2011 |
| 2012 |
| 2013 |
| 2014 |
| 2015 |
